# 9-[(Furan-2-ylmeth­yl)amino]-5-(3,4,5-trimeth­oxy­phen­yl)-5,5a,8a,9-tetra­hydro­furo[3′,4′:6,7]naphtho­[2,3-*d*][1,3]dioxol-6(8*H*)-one

**DOI:** 10.1107/S1600536811027735

**Published:** 2011-07-23

**Authors:** Gang Luo, Hong Chen, Jing Zhou, Dan-Li Tian, Shi Zhang

**Affiliations:** a School of Pharmacy, Tianjin Medical University, Tianjin 300070, People’s Republic of China; b Room of Pharmacognosy, Medical College of Chinese People’s Armed Police Forces, Tianjin 300162, People’s Republic of China; c Tianjin Key Laboratory for Biomarkers of Occupational, and Environmental Hazards, Tianjin 300162, People’s Republic of China

## Abstract

In title compound, C_27_H_27_NO_8_, the dihydrofuran-2(3*H*)-one ring and the six-membered ring fused to it both display envelope conformations. The dihedral angle between the benzene ring of the benzo[*d*][1,3]dioxole group and the other benzene ring is 60.59 (2)°. In the crystal, weak inter­molecular C—H⋯O hydrogen bonds link the mol­ecules into a three-dimensional network. The furan ring is disordered over two sets of sites with occupancies of 0.722 (7) and 0.278 (7)

## Related literature

For podophyllotoxin derivatives synthesized by our group, see: Lu *et al.* (2010 ▶); Yu *et al.* (2008 ▶); Zhao *et al.* (2009 ▶). For bond-length and angle data of related structures, see: Feng *et al.* (2008 ▶); Zhang *et al.* (1994 ▶); Zuo *et al.* (2009 ▶).
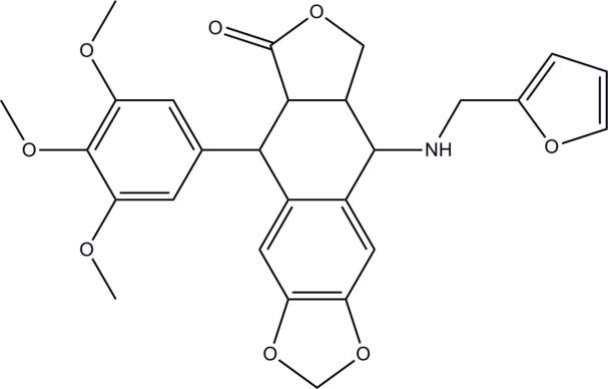


## Experimental

### 

#### Crystal data

C_27_H_27_NO_8_
*M* *_r_* = 493.50
Monoclinic, 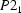

*a* = 11.7374 (15) Å
*b* = 8.4087 (8) Å
*c* = 11.8848 (16) Å
β = 102.693 (5)°
*V* = 1144.3 (2) Å^3^
*Z* = 2
Mo *K*α radiation
μ = 0.11 mm^−1^
*T* = 113 K
0.20 × 0.18 × 0.12 mm

#### Data collection

Rigaku Saturn724 CCD diffractometer
Absorption correction: multi-scan (*CrystalClear*; Rigaku, 2007 ▶) *T*_min_ = 0.979, *T*_max_ = 0.987
14991 measured reflections
2950 independent reflections
2262 reflections with *I* > 2σ(*I*)
*R*_int_ = 0.047

#### Refinement

*R*[*F* ^2^ > 2σ(*F* ^2^)] = 0.030
*wR*(*F* ^2^) = 0.067
*S* = 0.97
2950 reflections
372 parameters
41 restraints
H atoms treated by a mixture of independent and constrained refinement
Δρ_max_ = 0.17 e Å^−3^
Δρ_min_ = −0.22 e Å^−3^

### 

Data collection: *CrystalClear* (Rigaku, 2007 ▶); cell refinement: *CrystalClear*; data reduction: *CrystalClear*; program(s) used to solve structure: *SHELXS97* (Sheldrick, 2008 ▶); program(s) used to refine structure: *SHELXL97* (Sheldrick, 2008 ▶); molecular graphics: *SHELXTL* (Sheldrick, 2008 ▶); software used to prepare material for publication: *SHELXTL*.

## Supplementary Material

Crystal structure: contains datablock(s) I, global. DOI: 10.1107/S1600536811027735/rz2621sup1.cif

Structure factors: contains datablock(s) I. DOI: 10.1107/S1600536811027735/rz2621Isup2.hkl

Supplementary material file. DOI: 10.1107/S1600536811027735/rz2621Isup3.cml

Additional supplementary materials:  crystallographic information; 3D view; checkCIF report

## Figures and Tables

**Table 1:** Hydrogen-bond geometry (Å, °)

*D*—H⋯*A*	*D*—H	H⋯*A*	*D*⋯*A*	*D*—H⋯*A*
C25—H25*A*⋯O2^i^	0.98	2.60	3.268 (2)	126
C26—H26*C*⋯O3^ii^	0.98	2.60	3.544 (3)	163
C27—H27*A*⋯O1^iii^	0.98	2.54	3.487 (4)	164
C27—H27*B*⋯O7^iv^	0.98	2.50	2.993 (2)	111

Symmetry codes: (i) 
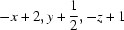
; (ii) 
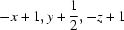
; (iii) 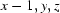; (iv) 
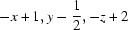
.

**Fig. 1.**
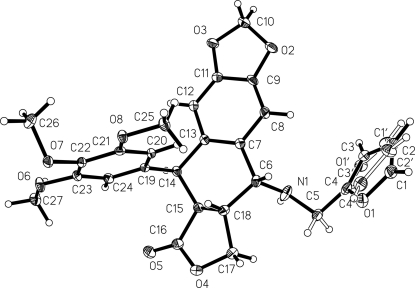
The molecular structure of the title compound, with displacement ellipsoids drawn at the 40% probability level.

## supplementary crystallographic information

### Comment

Podophyllotoxin and its derivatives are well known substances with anti-cancer activity. In recent years, our study has been aimed at synthesizing novel podophyllotoxin compounds with improved bioactivities (Lu *et al.*, 2010; Yu *et al.*, 2008; Zhao *et al.*, 2009). In this paper, we report the crystal structure of title the compound.

In title compound, bond lengths and angles are normal and in good agreement with those reported previously for related compounds (Feng *et al.*, 2008; Zhang, *et al.*, 1994; Zuo, *et al.*, 2009). The tetrahydrofuran ring (C15—C18/O4) and the six-membered ring (C6—C7/C13—C15/C18) fused to it both display envelope conformations. The dihedral angle between the benzene ring (C7—C9/C11—C13) of the benzo[*d*]-[1,3]dioxole fragment and the other benzene ring (C19—C24) is 60.59 (2)°. In the crystal structure, there are weak C—H···O intermolecular hydrogen interactions linking the molecules into a three-dimensional network (Table 1). The furan ring is disordered over two sets of sites with occupancies of 0.722 (7) and 0.278 (7).

### Experimental

The title compound was synthesized by two steps. A mixture of furan-2-carbaldehyde (0.115 g, 1.2 mmol), 4β-amino podophyllotoxin (0.420 g, 1 mmol) and two drops of acetic acid in 95% ethanol (30 ml) was stirred for 6 h, then an appropriate amount of NaBH_4_was added and the mixture stirred for 1 h at 273 K. After the addition of 5% HCl (5 ml) to end off the reaction, the mixture was concentrated *in vacuo* and the pH adjusted to basic conditions with a saturated NaHCO_3_ solution. The reaction mixture was extracted with CH_2_Cl_2_, dried over MgSO_4_ and concentrated *in vacuo*. The residue was dissolved in methanol and slow evaporation over two weeks at room temperature gave colourless crystals suitable for X-ray analysis

### Refinement

All H atoms bound to C atoms were found in a difference Fourier map, and included in the final cycles of refinement using a riding model, with C—H = 0.95–1.00 Å and with *U*_iso_(H) = 1.2*U*_eq_(C) for aryl and methylene H atoms and 1.5*U*_eq_(C) for the methyl H atoms. The amine H atom was refined freely. 690 Friedel pairs were merged. Similarity restraints in distances and thermal parameters were used in order to attain a reasonable geometry of the disordered furan ring.

### Crystal data

**Table:**

C_27_H_27_NO_8_	*F*(000) = 520
*M**_r_* = 493.50	*D*_x_ = 1.432 Mg m^−^^3^
Monoclinic, *P*2_1_	Mo *K*α radiation, λ = 0.71073 Å
Hall symbol: P 2yb	Cell parameters from 4290 reflections
*a* = 11.7374 (15) Å	θ = 1.8–28.0°
*b* = 8.4087 (8) Å	µ = 0.11 mm^−^^1^
*c* = 11.8848 (16) Å	*T* = 113 K
β = 102.693 (5)°	Prism, colourless
*V* = 1144.3 (2) Å^3^	0.20 × 0.18 × 0.12 mm
*Z* = 2	

### Data collection

**Table:**

Rigaku Saturn724 CCD diffractometer	2950 independent reflections
Radiation source: rotating anode	2262 reflections with *I* > 2σ(*I*)
multilayer	*R*_int_ = 0.047
Detector resolution: 14.22 pixels mm^-1^	θ_max_ = 28.0°, θ_min_ = 1.8°
ω and φ scans	*h* = −15→15
Absorption correction: multi-scan (*CrystalClear*; Rigaku, 2007)	*k* = −11→10
*T*_min_ = 0.979, *T*_max_ = 0.987	*l* = −15→15
14991 measured reflections	

### Refinement

**Table:**

Refinement on *F*^2^	Primary atom site location: structure-invariant direct methods
Least-squares matrix: full	Secondary atom site location: difference Fourier map
*R*[*F*^2^ > 2σ(*F*^2^)] = 0.030	Hydrogen site location: inferred from neighbouring sites
*wR*(*F*^2^) = 0.067	H atoms treated by a mixture of independent and constrained refinement
*S* = 0.97	*w* = 1/[σ^2^(*F*_o_^2^) + (0.036*P*)^2^] where *P* = (*F*_o_^2^ + 2*F*_c_^2^)/3
2950 reflections	(Δ/σ)_max_ = 0.001
372 parameters	Δρ_max_ = 0.17 e Å^−^^3^
41 restraints	Δρ_min_ = −0.22 e Å^−^^3^

### Special details

**Table:**

Geometry. All e.s.d.'s (except the e.s.d. in the dihedral angle between two l.s. planes) are estimated using the full covariance matrix. The cell e.s.d.'s are taken into account individually in the estimation of e.s.d.'s in distances, angles and torsion angles; correlations between e.s.d.'s in cell parameters are only used when they are defined by crystal symmetry. An approximate (isotropic) treatment of cell e.s.d.'s is used for estimating e.s.d.'s involving l.s. planes.
Refinement. Refinement of *F*^2^ against ALL reflections. The weighted *R*-factor *wR* and goodness of fit *S* are based on *F*^2^, conventional *R*-factors *R* are based on *F*, with *F* set to zero for negative *F*^2^. The threshold expression of *F*^2^ > σ(*F*^2^) is used only for calculating *R*-factors(gt) *etc*. and is not relevant to the choice of reflections for refinement. *R*-factors based on *F*^2^ are statistically about twice as large as those based on *F*, and *R*- factors based on ALL data will be even larger.

### Fractional atomic coordinates and isotropic or equivalent isotropic displacement parameters (Å^2^)

**Table:**

	*x*	*y*	*z*	*U*_iso_*/*U*_eq_	Occ. (<1)
O1	1.3701 (3)	0.9258 (3)	0.7345 (3)	0.0259 (8)	0.722 (7)
C1	1.4119 (3)	0.9416 (4)	0.6362 (3)	0.0258 (9)	0.722 (7)
H1A	1.4695	1.0165	0.6263	0.031*	0.722 (7)
C2	1.3609 (4)	0.8374 (6)	0.5562 (4)	0.0255 (13)	0.722 (7)
H2	1.3750	0.8249	0.4810	0.031*	0.722 (7)
C3	1.2805 (3)	0.7482 (5)	0.6062 (3)	0.0225 (8)	0.722 (7)
H3	1.2312	0.6647	0.5703	0.027*	0.722 (7)
C4	1.2879 (4)	0.8046 (6)	0.7136 (4)	0.0219 (5)	0.722 (7)
O1'	1.2713 (6)	0.6797 (10)	0.6369 (6)	0.029 (2)	0.278 (7)
C1'	1.3229 (8)	0.7386 (14)	0.5538 (7)	0.032 (3)	0.278 (7)
H1'	1.3261	0.6824	0.4852	0.038*	0.278 (7)
C2'	1.3680 (11)	0.8821 (13)	0.5781 (10)	0.034 (5)	0.278 (7)
H2'	1.4078	0.9450	0.5325	0.040*	0.278 (7)
C3'	1.3437 (11)	0.9230 (11)	0.6900 (10)	0.027 (3)	0.278 (7)
H3'	1.3639	1.0178	0.7332	0.033*	0.278 (7)
C4'	1.2882 (10)	0.8003 (14)	0.7168 (9)	0.0219 (5)	0.278 (7)
O2	0.86595 (12)	0.7908 (2)	0.34547 (11)	0.0321 (4)	
O3	0.67662 (11)	0.79787 (18)	0.36877 (11)	0.0236 (3)	
O4	1.00549 (13)	0.9413 (2)	1.06166 (11)	0.0362 (4)	
O5	0.81900 (13)	0.8872 (2)	1.05584 (13)	0.0388 (4)	
O6	0.52676 (11)	1.28410 (17)	0.92051 (12)	0.0234 (3)	
O7	0.62669 (11)	1.52498 (15)	0.83580 (11)	0.0185 (3)	
O8	0.79077 (11)	1.47895 (16)	0.71823 (11)	0.0174 (3)	
N1	1.10768 (16)	0.7428 (2)	0.77354 (18)	0.0272 (4)	
C5	1.23354 (16)	0.7632 (3)	0.81295 (17)	0.0244 (5)	
H5A	1.2492	0.8466	0.8694	0.029*	0.722 (7)
H5B	1.2673	0.6666	0.8485	0.029*	0.722 (7)
H5C	1.2489	0.8474	0.8688	0.029*	0.278 (7)
H5D	1.2669	0.6670	0.8495	0.029*	0.278 (7)
C6	1.04161 (16)	0.8907 (2)	0.75745 (16)	0.0186 (4)	
H6	1.0923	0.9741	0.7336	0.022*	
C7	0.93416 (15)	0.8704 (2)	0.65914 (16)	0.0164 (4)	
C8	0.95707 (17)	0.8448 (3)	0.54918 (16)	0.0216 (4)	
H8	1.0349	0.8434	0.5385	0.026*	
C9	0.86540 (16)	0.8223 (2)	0.45889 (16)	0.0211 (4)	
C10	0.74725 (18)	0.8030 (3)	0.28551 (17)	0.0291 (5)	
H10A	0.7269	0.7138	0.2304	0.035*	
H10B	0.7343	0.9040	0.2419	0.035*	
C11	0.75225 (16)	0.8251 (2)	0.47291 (16)	0.0182 (4)	
C12	0.72683 (16)	0.8476 (2)	0.57774 (16)	0.0168 (4)	
H12	0.6483	0.8477	0.5863	0.020*	
C13	0.81992 (16)	0.8708 (2)	0.67332 (16)	0.0159 (4)	
C14	0.78895 (16)	0.8993 (2)	0.78943 (16)	0.0164 (4)	
H14	0.7264	0.8221	0.7974	0.020*	
C15	0.89755 (16)	0.8617 (2)	0.88232 (16)	0.0191 (4)	
H15	0.9140	0.7457	0.8766	0.023*	
C16	0.89706 (19)	0.8959 (3)	1.00600 (18)	0.0283 (5)	
C17	1.08442 (18)	0.9333 (3)	0.98287 (17)	0.0280 (5)	
H17A	1.1261	0.8302	0.9899	0.034*	
H17B	1.1424	1.0205	0.9980	0.034*	
C18	1.00434 (16)	0.9508 (3)	0.86503 (16)	0.0200 (4)	
H18	0.9831	1.0657	0.8531	0.024*	
C19	0.74248 (16)	1.0657 (2)	0.79825 (15)	0.0147 (4)	
C20	0.78823 (15)	1.1934 (2)	0.74867 (16)	0.0151 (4)	
H20	0.8466	1.1755	0.7060	0.018*	
C21	0.74977 (15)	1.3459 (2)	0.76073 (15)	0.0148 (4)	
C22	0.66214 (15)	1.3726 (2)	0.81994 (16)	0.0151 (4)	
C23	0.61405 (15)	1.2451 (2)	0.86715 (16)	0.0166 (4)	
C24	0.65474 (16)	1.0917 (2)	0.85763 (16)	0.0164 (4)	
H24	0.6227	1.0050	0.8916	0.020*	
C25	0.88269 (16)	1.4550 (2)	0.65900 (16)	0.0182 (4)	
H25A	0.9504	1.4086	0.7120	0.027*	
H25B	0.9048	1.5573	0.6304	0.027*	
H25C	0.8561	1.3827	0.5938	0.027*	
C26	0.54369 (17)	1.5820 (3)	0.73793 (18)	0.0254 (5)	
H26A	0.5794	1.5843	0.6708	0.038*	
H26B	0.5190	1.6896	0.7536	0.038*	
H26C	0.4757	1.5113	0.7222	0.038*	
C27	0.47759 (17)	1.1602 (3)	0.97667 (17)	0.0227 (5)	
H27A	0.4453	1.0777	0.9204	0.034*	
H27B	0.4153	1.2039	1.0104	0.034*	
H27C	0.5384	1.1139	1.0378	0.034*	
H1	1.086 (2)	0.676 (4)	0.803 (2)	0.056 (10)*	

### Atomic displacement parameters (Å^2^)

**Table:**

	*U*^11^	*U*^22^	*U*^33^	*U*^12^	*U*^13^	*U*^23^
O1	0.0233 (16)	0.0261 (14)	0.0294 (18)	0.0016 (11)	0.0081 (13)	−0.0019 (12)
C1	0.0237 (17)	0.0275 (18)	0.0266 (19)	0.0037 (14)	0.0063 (15)	0.0045 (16)
C2	0.021 (2)	0.031 (3)	0.023 (2)	0.003 (2)	0.0028 (15)	0.004 (2)
C3	0.0174 (17)	0.029 (2)	0.020 (2)	0.0030 (17)	0.0016 (14)	0.0017 (17)
C4	0.0147 (9)	0.0226 (12)	0.0261 (11)	0.0049 (9)	−0.0004 (8)	−0.0031 (9)
O1'	0.027 (3)	0.037 (5)	0.022 (4)	0.004 (3)	0.002 (3)	−0.005 (3)
C1'	0.030 (5)	0.040 (6)	0.024 (4)	0.010 (5)	0.002 (4)	0.009 (4)
C2'	0.037 (7)	0.044 (8)	0.022 (6)	0.004 (5)	0.010 (5)	0.008 (6)
C3'	0.031 (5)	0.019 (5)	0.031 (6)	0.009 (4)	0.007 (5)	0.003 (4)
C4'	0.0147 (9)	0.0226 (12)	0.0261 (11)	0.0049 (9)	−0.0004 (8)	−0.0031 (9)
O2	0.0278 (8)	0.0550 (11)	0.0151 (7)	−0.0014 (8)	0.0079 (6)	−0.0012 (7)
O3	0.0236 (7)	0.0315 (9)	0.0156 (7)	−0.0025 (7)	0.0038 (6)	−0.0010 (6)
O4	0.0300 (8)	0.0597 (12)	0.0172 (7)	0.0130 (8)	0.0012 (7)	−0.0025 (8)
O5	0.0378 (9)	0.0615 (12)	0.0202 (8)	0.0147 (8)	0.0132 (7)	0.0081 (8)
O6	0.0262 (7)	0.0208 (8)	0.0295 (8)	0.0029 (6)	0.0195 (6)	0.0037 (6)
O7	0.0215 (7)	0.0163 (7)	0.0186 (7)	0.0047 (6)	0.0065 (6)	0.0007 (6)
O8	0.0189 (7)	0.0149 (7)	0.0210 (7)	0.0012 (5)	0.0102 (6)	0.0022 (6)
N1	0.0166 (9)	0.0188 (10)	0.0440 (12)	0.0032 (8)	0.0019 (8)	0.0030 (9)
C5	0.0178 (10)	0.0288 (13)	0.0250 (11)	0.0052 (9)	0.0011 (8)	−0.0034 (9)
C6	0.0180 (9)	0.0163 (10)	0.0228 (10)	0.0021 (8)	0.0069 (8)	0.0021 (8)
C7	0.0190 (9)	0.0124 (9)	0.0187 (10)	0.0009 (8)	0.0062 (8)	0.0028 (8)
C8	0.0188 (9)	0.0261 (12)	0.0223 (10)	0.0008 (9)	0.0095 (8)	0.0043 (9)
C9	0.0269 (11)	0.0237 (11)	0.0154 (10)	0.0002 (9)	0.0107 (8)	0.0026 (9)
C10	0.0290 (11)	0.0415 (14)	0.0175 (10)	0.0022 (11)	0.0067 (9)	−0.0018 (10)
C11	0.0208 (10)	0.0160 (10)	0.0174 (9)	−0.0011 (8)	0.0031 (8)	0.0010 (8)
C12	0.0170 (9)	0.0144 (10)	0.0198 (10)	−0.0001 (8)	0.0062 (8)	0.0006 (8)
C13	0.0211 (9)	0.0107 (9)	0.0171 (9)	0.0019 (8)	0.0070 (8)	0.0019 (8)
C14	0.0181 (9)	0.0157 (10)	0.0170 (9)	0.0003 (8)	0.0070 (7)	0.0009 (8)
C15	0.0233 (10)	0.0188 (10)	0.0157 (9)	0.0058 (9)	0.0058 (8)	0.0035 (8)
C16	0.0304 (11)	0.0353 (13)	0.0189 (10)	0.0121 (11)	0.0050 (9)	0.0056 (10)
C17	0.0235 (11)	0.0377 (13)	0.0217 (10)	0.0083 (10)	0.0027 (9)	−0.0007 (10)
C18	0.0201 (10)	0.0217 (11)	0.0173 (9)	0.0040 (9)	0.0016 (8)	0.0019 (9)
C19	0.0152 (9)	0.0163 (10)	0.0122 (9)	0.0011 (8)	0.0020 (7)	−0.0005 (8)
C20	0.0132 (8)	0.0187 (10)	0.0139 (9)	0.0017 (8)	0.0042 (7)	0.0000 (7)
C21	0.0143 (8)	0.0178 (10)	0.0119 (9)	−0.0009 (8)	0.0017 (7)	0.0029 (8)
C22	0.0167 (9)	0.0144 (9)	0.0145 (9)	0.0035 (8)	0.0039 (7)	−0.0002 (8)
C23	0.0161 (9)	0.0207 (11)	0.0150 (9)	0.0015 (8)	0.0073 (7)	0.0005 (8)
C24	0.0179 (10)	0.0165 (10)	0.0155 (9)	−0.0018 (8)	0.0054 (8)	0.0016 (8)
C25	0.0186 (9)	0.0177 (10)	0.0212 (10)	−0.0037 (8)	0.0103 (8)	0.0001 (9)
C26	0.0224 (11)	0.0200 (11)	0.0323 (12)	0.0059 (9)	0.0030 (9)	0.0037 (9)
C27	0.0225 (11)	0.0262 (12)	0.0232 (11)	−0.0024 (9)	0.0129 (9)	0.0033 (9)

### Geometric parameters (Å, °)

**Table:**

O1—C1	1.370 (4)	C6—H6	1.0000
O1—C4	1.388 (5)	C7—C13	1.388 (2)
C1—C2	1.334 (5)	C7—C8	1.407 (2)
C1—H1A	0.9500	C8—C9	1.356 (3)
C2—C3	1.432 (6)	C8—H8	0.9500
C2—H2	0.9500	C9—C11	1.375 (2)
C3—C4	1.346 (5)	C10—H10A	0.9900
C3—H3	0.9500	C10—H10B	0.9900
C4—C5	1.502 (4)	C11—C12	1.357 (3)
O1'—C1'	1.360 (8)	C12—C13	1.406 (2)
O1'—C4'	1.374 (9)	C12—H12	0.9500
C1'—C2'	1.324 (9)	C13—C14	1.521 (2)
C1'—H1'	0.9500	C14—C19	1.514 (3)
C2'—C3'	1.461 (9)	C14—C15	1.525 (3)
C2'—H2'	0.9500	C14—H14	1.0000
C3'—C4'	1.297 (9)	C15—C16	1.499 (3)
C3'—H3'	0.9500	C15—C18	1.512 (3)
C4'—C5	1.461 (9)	C15—H15	1.0000
O2—C9	1.375 (2)	C17—C18	1.513 (3)
O2—C10	1.422 (2)	C17—H17A	0.9900
O3—C11	1.374 (2)	C17—H17B	0.9900
O3—C10	1.424 (2)	C18—H18	1.0000
O4—C16	1.354 (3)	C19—C24	1.388 (3)
O4—C17	1.456 (2)	C19—C20	1.389 (3)
O5—C16	1.198 (2)	C20—C21	1.377 (3)
O6—C23	1.358 (2)	C20—H20	0.9500
O6—C27	1.426 (2)	C21—C22	1.386 (2)
O7—C22	1.373 (2)	C22—C23	1.387 (3)
O7—C26	1.426 (2)	C23—C24	1.389 (3)
O8—C21	1.358 (2)	C24—H24	0.9500
O8—C25	1.426 (2)	C25—H25A	0.9800
N1—C6	1.456 (3)	C25—H25B	0.9800
N1—C5	1.458 (2)	C25—H25C	0.9800
N1—H1	0.74 (3)	C26—H26A	0.9800
C5—H5A	0.9601	C26—H26B	0.9800
C5—H5B	0.9600	C26—H26C	0.9800
C5—H5C	0.9600	C27—H27A	0.9800
C5—H5D	0.9600	C27—H27B	0.9800
C6—C18	1.525 (3)	C27—H27C	0.9800
C6—C7	1.529 (3)		
			
C1—O1—C4	106.4 (3)	H10A—C10—H10B	108.5
C2—C1—O1	110.8 (3)	C12—C11—O3	128.41 (17)
C2—C1—H1A	124.6	C12—C11—C9	121.80 (18)
O1—C1—H1A	124.6	O3—C11—C9	109.76 (16)
C1—C2—C3	106.4 (4)	C11—C12—C13	118.20 (17)
C1—C2—H2	126.8	C11—C12—H12	120.9
C3—C2—H2	126.8	C13—C12—H12	120.9
C4—C3—C2	107.3 (3)	C7—C13—C12	120.12 (17)
C4—C3—H3	126.3	C7—C13—C14	122.71 (17)
C2—C3—H3	126.3	C12—C13—C14	117.16 (16)
C3—C4—O1	109.1 (3)	C19—C14—C13	111.73 (15)
C3—C4—C5	135.6 (4)	C19—C14—C15	113.45 (16)
O1—C4—C5	115.3 (4)	C13—C14—C15	107.20 (15)
C1'—O1'—C4'	102.4 (6)	C19—C14—H14	108.1
C2'—C1'—O1'	113.2 (8)	C13—C14—H14	108.1
C2'—C1'—H1'	123.4	C15—C14—H14	108.1
O1'—C1'—H1'	123.4	C16—C15—C18	102.73 (17)
C1'—C2'—C3'	105.3 (7)	C16—C15—C14	119.19 (16)
C1'—C2'—H2'	127.3	C18—C15—C14	112.65 (16)
C3'—C2'—H2'	127.3	C16—C15—H15	107.2
C4'—C3'—C2'	104.3 (7)	C18—C15—H15	107.2
C4'—C3'—H3'	127.9	C14—C15—H15	107.2
C2'—C3'—H3'	127.9	O5—C16—O4	120.97 (19)
C3'—C4'—O1'	114.9 (8)	O5—C16—C15	129.9 (2)
C3'—C4'—C5	133.9 (10)	O4—C16—C15	109.12 (17)
O1'—C4'—C5	111.2 (8)	O4—C17—C18	103.72 (15)
C9—O2—C10	105.20 (15)	O4—C17—H17A	111.0
C11—O3—C10	105.14 (14)	C18—C17—H17A	111.0
C16—O4—C17	109.59 (16)	O4—C17—H17B	111.0
C23—O6—C27	117.80 (15)	C18—C17—H17B	111.0
C22—O7—C26	112.03 (15)	H17A—C17—H17B	109.0
C21—O8—C25	115.83 (14)	C15—C18—C17	101.01 (16)
C6—N1—C5	114.47 (17)	C15—C18—C6	110.41 (17)
C6—N1—H1	119 (2)	C17—C18—C6	120.64 (16)
C5—N1—H1	112 (2)	C15—C18—H18	108.0
N1—C5—C4'	111.2 (5)	C17—C18—H18	108.0
N1—C5—C4	110.5 (2)	C6—C18—H18	108.0
N1—C5—H5A	109.6	C24—C19—C20	119.56 (17)
C4'—C5—H5A	109.9	C24—C19—C14	119.99 (16)
C4—C5—H5A	109.4	C20—C19—C14	120.45 (16)
N1—C5—H5B	109.4	C21—C20—C19	120.65 (17)
C4'—C5—H5B	108.4	C21—C20—H20	119.7
C4—C5—H5B	109.7	C19—C20—H20	119.7
H5A—C5—H5B	108.3	O8—C21—C20	125.24 (16)
N1—C5—H5C	109.4	O8—C21—C22	114.73 (17)
C4'—C5—H5C	109.6	C20—C21—C22	120.03 (17)
C4—C5—H5C	109.0	O7—C22—C21	120.13 (17)
H5B—C5—H5C	108.8	O7—C22—C23	120.24 (16)
N1—C5—H5D	109.3	C21—C22—C23	119.57 (17)
C4'—C5—H5D	109.2	O6—C23—C22	114.70 (17)
C4—C5—H5D	110.5	O6—C23—C24	124.80 (17)
H5A—C5—H5D	107.6	C22—C23—C24	120.50 (16)
H5C—C5—H5D	108.2	C19—C24—C23	119.65 (17)
N1—C6—C18	114.55 (17)	C19—C24—H24	120.2
N1—C6—C7	109.34 (16)	C23—C24—H24	120.2
C18—C6—C7	109.71 (14)	O8—C25—H25A	109.5
N1—C6—H6	107.7	O8—C25—H25B	109.5
C18—C6—H6	107.7	H25A—C25—H25B	109.5
C7—C6—H6	107.7	O8—C25—H25C	109.5
C13—C7—C8	120.00 (17)	H25A—C25—H25C	109.5
C13—C7—C6	124.32 (16)	H25B—C25—H25C	109.5
C8—C7—C6	115.65 (16)	O7—C26—H26A	109.5
C9—C8—C7	118.42 (17)	O7—C26—H26B	109.5
C9—C8—H8	120.8	H26A—C26—H26B	109.5
C7—C8—H8	120.8	O7—C26—H26C	109.5
C8—C9—C11	121.44 (18)	H26A—C26—H26C	109.5
C8—C9—O2	128.97 (17)	H26B—C26—H26C	109.5
C11—C9—O2	109.57 (17)	O6—C27—H27A	109.5
O2—C10—O3	107.78 (15)	O6—C27—H27B	109.5
O2—C10—H10A	110.2	H27A—C27—H27B	109.5
O3—C10—H10A	110.2	O6—C27—H27C	109.5
O2—C10—H10B	110.2	H27A—C27—H27C	109.5
O3—C10—H10B	110.2	H27B—C27—H27C	109.5
			
C4—O1—C1—C2	−0.1 (2)	C7—C13—C14—C19	103.5 (2)
O1—C1—C2—C3	0.0 (2)	C12—C13—C14—C19	−75.1 (2)
C1—C2—C3—C4	0.1 (3)	C7—C13—C14—C15	−21.3 (2)
C2—C3—C4—O1	−0.2 (4)	C12—C13—C14—C15	160.05 (17)
C2—C3—C4—C5	−177.2 (5)	C19—C14—C15—C16	49.7 (2)
C1—O1—C4—C3	0.2 (3)	C13—C14—C15—C16	173.51 (18)
C1—O1—C4—C5	177.9 (3)	C19—C14—C15—C18	−70.7 (2)
C4'—O1'—C1'—C2'	0.0 (3)	C13—C14—C15—C18	53.1 (2)
O1'—C1'—C2'—C3'	0.0 (3)	C17—O4—C16—O5	176.9 (2)
C1'—C2'—C3'—C4'	0.0 (5)	C17—O4—C16—C15	−2.7 (2)
C2'—C3'—C4'—O1'	0.0 (7)	C18—C15—C16—O5	160.0 (2)
C2'—C3'—C4'—C5	−176.7 (12)	C14—C15—C16—O5	34.7 (4)
C1'—O1'—C4'—C3'	0.0 (6)	C18—C15—C16—O4	−20.5 (2)
C1'—O1'—C4'—C5	177.4 (9)	C14—C15—C16—O4	−145.80 (18)
C6—N1—C5—C4'	−81.7 (5)	C16—O4—C17—C18	24.8 (2)
C6—N1—C5—C4	−80.5 (3)	C16—C15—C18—C17	33.8 (2)
C3'—C4'—C5—N1	115.8 (10)	C14—C15—C18—C17	163.25 (17)
O1'—C4'—C5—N1	−61.0 (8)	C16—C15—C18—C6	162.55 (16)
C3'—C4'—C5—C4	58 (30)	C14—C15—C18—C6	−68.0 (2)
O1'—C4'—C5—C4	−119 (30)	O4—C17—C18—C15	−35.8 (2)
C3—C4—C5—N1	−47.2 (6)	O4—C17—C18—C6	−157.70 (18)
O1—C4—C5—N1	136.0 (3)	N1—C6—C18—C15	−79.7 (2)
C3—C4—C5—C4'	75 (30)	C7—C6—C18—C15	43.7 (2)
O1—C4—C5—C4'	−102 (30)	N1—C6—C18—C17	37.5 (3)
C5—N1—C6—C18	−88.4 (2)	C7—C6—C18—C17	160.88 (19)
C5—N1—C6—C7	148.05 (17)	C13—C14—C19—C24	144.26 (17)
N1—C6—C7—C13	112.7 (2)	C15—C14—C19—C24	−94.4 (2)
C18—C6—C7—C13	−13.7 (3)	C13—C14—C19—C20	−36.7 (2)
N1—C6—C7—C8	−65.2 (2)	C15—C14—C19—C20	84.6 (2)
C18—C6—C7—C8	168.40 (17)	C24—C19—C20—C21	1.9 (3)
C13—C7—C8—C9	0.5 (3)	C14—C19—C20—C21	−177.17 (18)
C6—C7—C8—C9	178.49 (19)	C25—O8—C21—C20	−1.4 (2)
C7—C8—C9—C11	0.5 (3)	C25—O8—C21—C22	178.75 (15)
C7—C8—C9—O2	−177.89 (19)	C19—C20—C21—O8	178.27 (17)
C10—O2—C9—C8	−171.2 (2)	C19—C20—C21—C22	−1.9 (3)
C10—O2—C9—C11	10.3 (2)	C26—O7—C22—C21	83.0 (2)
C9—O2—C10—O3	−15.8 (2)	C26—O7—C22—C23	−99.73 (19)
C11—O3—C10—O2	15.3 (2)	O8—C21—C22—O7	−2.6 (2)
C10—O3—C11—C12	172.9 (2)	C20—C21—C22—O7	177.56 (16)
C10—O3—C11—C9	−9.0 (2)	O8—C21—C22—C23	−179.89 (16)
C8—C9—C11—C12	−1.2 (3)	C20—C21—C22—C23	0.3 (3)
O2—C9—C11—C12	177.42 (18)	C27—O6—C23—C22	−176.57 (16)
C8—C9—C11—O3	−179.50 (19)	C27—O6—C23—C24	3.8 (3)
O2—C9—C11—O3	−0.9 (2)	O7—C22—C23—O6	4.4 (2)
O3—C11—C12—C13	178.88 (18)	C21—C22—C23—O6	−178.29 (16)
C9—C11—C12—C13	0.9 (3)	O7—C22—C23—C24	−175.91 (18)
C8—C7—C13—C12	−0.8 (3)	C21—C22—C23—C24	1.4 (3)
C6—C7—C13—C12	−178.56 (18)	C20—C19—C24—C23	−0.2 (3)
C8—C7—C13—C14	−179.37 (18)	C14—C19—C24—C23	178.83 (17)
C6—C7—C13—C14	2.9 (3)	O6—C23—C24—C19	178.24 (17)
C11—C12—C13—C7	0.1 (3)	C22—C23—C24—C19	−1.4 (3)
C11—C12—C13—C14	178.72 (17)		

### Hydrogen-bond geometry (Å, °)

**Table:**

*D*—H···*A*	*D*—H	H···*A*	*D*···*A*	*D*—H···*A*
C25—H25A···O2^i^	0.98	2.60	3.268 (2)	126.
C26—H26C···O3^ii^	0.98	2.60	3.544 (3)	163.
C27—H27A···O1^iii^	0.98	2.54	3.487 (4)	164.
C27—H27B···O7^iv^	0.98	2.50	2.993 (2)	111.

Symmetry codes: (i) −*x*+2, *y*+1/2, −*z*+1; (ii) −*x*+1, *y*+1/2, −*z*+1; (iii) *x*−1, *y*, *z*; (iv) −*x*+1, *y*−1/2, −*z*+2.
